# Malnutrition in older adults with cancer undergoing outpatient care: associated factors

**DOI:** 10.1007/s00520-026-10388-5

**Published:** 2026-03-06

**Authors:** Fernanda Rafaella de Melo Silva, Jurema Telles de Oliveira Lima Sales, Gabriel de Morais Borba, Ana Carolina Resende Silveira, Maria Júlia Gonçalves de Mello, Ana Paula Trussardi Fayh, Alex Sandro Rolland Souza

**Affiliations:** 1 Instituto de Medicina Integral Prof. Fernando Figueira (IMIP), Recife, PE Brazil; 2 Faculdade Pernambucana de Saúde (FPS), Recife, PE Brazil; 3 Federal University of Rio Grande Do Norte (UFRN), Natal, RN Brazil; 4 Federal University of Pernambuco (UFPE), Recife, PE Brazil; 5 Catholic University of Pernambuco (UNICAP), Recife, PE Brazil; 6 Natal, Brazil

**Keywords:** Cancer, Aged, Ambulatory care, Malnutrition, Risk factors

## Abstract

**Objectives:**

To determine the prevalence and factors associated with malnutrition in older adults with cancer upon admission for outpatient treatment.

**Methods:**

This cross-sectional study was conducted with older adults with cancer admitted to an oncogeriatrics outpatient clinic from 2015 to 2020 in the Northeast of Brazil. Sociodemographic data, lifestyle, and clinical variables were collected. Nutritional status was assessed using the Mini Nutritional Assessment short-form (MNA-SF) and classified as normal nutritional status (12 to 14 points), at risk of malnutrition (8 to 11 points), or malnourished (0 to 7 points). The multivariate Poisson regression was used to verify the association between the independent variables and malnutrition.

**Results:**

A total of 1954 patients were included. Of these, 14.9% were at risk of malnutrition, and 31.5% were malnourished. The risk factors for malnutrition were female gender (prevalence ratio [PR] = 1.28; 95% confidence interval [CI] = 1.03–1.59), upper gastrointestinal tumor (PR = 2.39; 95%CI = 1.66–3.45), colon, rectum, anus, and anal canal tumors (PR = 2.54; 95%CI = 1.77–3.64), lung tumor (PR = 2.35; 95%CI = 1.37–4.02), metastasis (PR = 1.37; 95%CI = 1.11–1.70), history of falls (PR = 1.27; 95%CI = 1.01–1.61), sedentary lifestyle (PR = 1.46; 95%CI = 1.11–1.93), and risk of depression (PR = 1.42; 95%CI = 1.16–1.73).

**Conclusion:**

The prevalence of nutritional risk and malnutrition was relatively high in older adults with cancer at the beginning of outpatient treatment. These findings underscore the need for routine malnutrition screening at admission in oncology outpatient settings to ensure early identification and management. The associated factors were easily identifiable within standard clinical evaluations, supporting the feasibility of systematic screening.

## Introduction

Older adults have a greater susceptibility to malnutrition due to physiological changes related to aging. One of these changes is reduced appetite, which increases the risk of adverse health outcomes and contributes to the development of geriatric syndromes, such as sarcopenia and frailty [1, 2]. Additionally, low income, limited social support, food insecurity, reduced functional capacity, and comorbidities are factors that heighten the nutritional risk in this population [2, 3].

The prevalence of malnutrition is higher in older adults with cancer, varying from 25 to 87% [3–6]. Malnutrition risk is worsened by the type and stage of the tumor and the side effects of cancer treatment, especially those associated with reduced food intake (e.g., anorexia, nausea, vomiting, dyspepsia, and diarrhea) [3, 4, 7, 8]. Prolonged fasting for examinations, reduced functional capacity, polypharmacy, dementia, depression, and inadequate nutritional support since the diagnosis also contribute to the nutritional risk [3, 4, 7].

Thus, malnutrition may impair the cancer treatment of older adults, increasing the recovery time [7, 9] and also leading to a worse prognosis with possible post-operative complications, greater risks of hospitalization and readmission risks, longer hospitalization length of stay, infections, mortality, and poor quality of life [3, 5, 6, 10]. Considering that these aspects elevate healthcare costs and 20% of deaths among patients with cancer are due to malnutrition complications, the early identification of this condition must be a priority in oncology care centers [11–13].

International guidelines on nutrition [14–16], the National Cancer Institute [12], and the International Society of Geriatric Oncology [17] recommend the use of practical, cheap, and replicable tools that ensure a timely diagnosis of malnutrition. Triage and nutritional evaluation tools can be useful in identifying malnutrition in patients with cancer. In addition, the main guidelines agree that the Mini Nutritional Assessment (MNA) is a viable and often-used tool to evaluate the nutritional status of older adults [12, 14–17].

The MNA verifies the nutritional status in multiple healthcare settings based on anthropometric measurements and dietary, psychological, medication, physical, and self-perceived health evaluations [18]. Thus, MNA presents good accuracy in identifying malnutrition in older adults with cancer [19]. In addition, previous studies showed that malnutrition, assessed by MNA, was associated with mortality, infection, hospitalization, and prolonged hospital length of stay [3, 5, 6].

Older adults with cancer present a greater nutritional risk that may lead to clinical worsening. Considering that the assessment of nutritional status must be included in the initial evaluation before oncological treatment, this study used the MNA-SF due to its practicality and efficiency. The MNA-SF is a validated, sensitive short-form tool that provides a reliable nutritional diagnosis using fewer items than the full MNA, making it particularly suitable for routine assessments in busy outpatient oncology settings. Therefore, this study aimed to verify the prevalence and factors associated with malnutrition in older adults with cancer upon admission for outpatient treatment using the MNA-SF.

## Methods and materials

### Study design, place, and cohort

This cross-sectional study used the baseline data from a cohort study [6] that included older adults with cancer followed up at the oncogeriatrics outpatient clinic of the *Instituto de Medicina Integral Prof. Fernando Figueira* (IMIP), a high-complexity oncology care center in Recife, Pernambuco, in the Northeast of Brazil. Data were collected between January 2015 and August 2020, and the study began after approval by the research ethics committee of the IMIP.

Inclusion criteria considered older adults aged ≥ 60 years [20] diagnosed with cancer (confirmed by histology, cytology, or immunohistochemistry). Those with skin cancer (e.g., basal cell carcinoma or non-metastatic epidermoid carcinoma), hematological cancer, subjected to previous non-surgical oncological treatment, or with other diseases (e.g., congestive cardiac failure, chronic kidney disease, liver failure, chronic obstructive pulmonary disease, or AIDS) were excluded.

The sample size (1397 patients) was calculated using the software STATA 13.1SE (Stata Corporation, College Station, TX, USA), with a 95% confidence level, error margin of 2.5%, and a malnutrition prevalence of ≤ 35% [5].

### Evaluations

A multiprofessional assessment at the time of admission to the outpatient clinic was conducted by a team composed of a clinical oncologist, geriatrician, nutritionist, physical therapist, physical educator, speech therapist, and occupational therapist. According to the institutional protocol, data were collected on sociodemographic, socioeconomic, clinical variables, and the Comprehensive Geriatric Assessment (CGA).

The independent variables encompassed sociodemographic (age [years], gender [female or male], family income [minimum wage at the moment of data collection], lifestyle (alcohol consumption, smoking, and level of physical activity [21]), and clinical data (primary cancer site, metastasis, comorbidities [22], functionality [23], polypharmacy [24], risk of depression [25], and history of falls in the last year).

The level of physical activity was determined by the International Physical Activity Questionnaire (short version), which classifies an individual as very active, active, moderately active, or sedentary (i.e., a person who practices less than 10 min of physical activity during the last seven days) [21]. The Charlson Comorbidity Index defined the presence of comorbidity when more than two comorbidities were present; cancer was excluded because it was common to all patients [22]. The functionality was assessed by the Karnofsky Performance Scale, which was considered reduced if ≤ 50% [23]. Polypharmacy was defined as the use of five or more medicaments [24], and the Geriatric Depression Scale evaluated the risk of depression; patients were considered depressed when they scored five or more points [25].

To obtain the anthropometric variables (weight and height), each measurement was taken twice; if the two values differed, a third measurement was performed, and the final value was recorded as the average of the two closest measurements [26]. Weight and height were measured using a fixed vertical digital platform scale with an attached stadiometer (Welmy® W200/100, São Paulo, Brazil), with a variation of 100 g, 200 kg capacity, and 0.5 cm precision. Body mass index (BMI) was calculated as weight/height^2^ and classified according to the Pan American Health Organization (PAHO)/WHO recommendations for older adults [26, 27].

Nutritional status was assessed using MNA-SF, which identifies older adults at risk of malnutrition or malnourished. This tool uses six questions to evaluate changes in appetite, involuntary weight loss, mobility, psychological stress, acute or neuropsychological diseases, and body mass index [18, 28]. Nutritional status was classified as normal nutritional status (12 to 14 points), at risk of malnutrition (i.e., exposure to factors that may lead to malnutrition; 8 to 11 points), or malnourished (0 to 7 points) [28].

### Statistical analysis

Categorical data were described using absolute and relative frequencies. The Shapiro-Wilk test was used to verify the data normality of continuous variables, which were presented using measures of central tendency and dispersion. The Pearson’s chi-square test was used to compare the nutritional status. The association between independent variables and nutritional status was analyzed using the multivariate Poisson regression. All variables with a *p*-value < 0.20 were included in the multivariate model to adjust the effects of independent predictors, adopting a significance level of 5%, a 95% confidence interval (95%CI), and calculating the prevalence ratio (PR). All data were analyzed using the STATA 13.1SE.

## Results

**Fig. 1:**
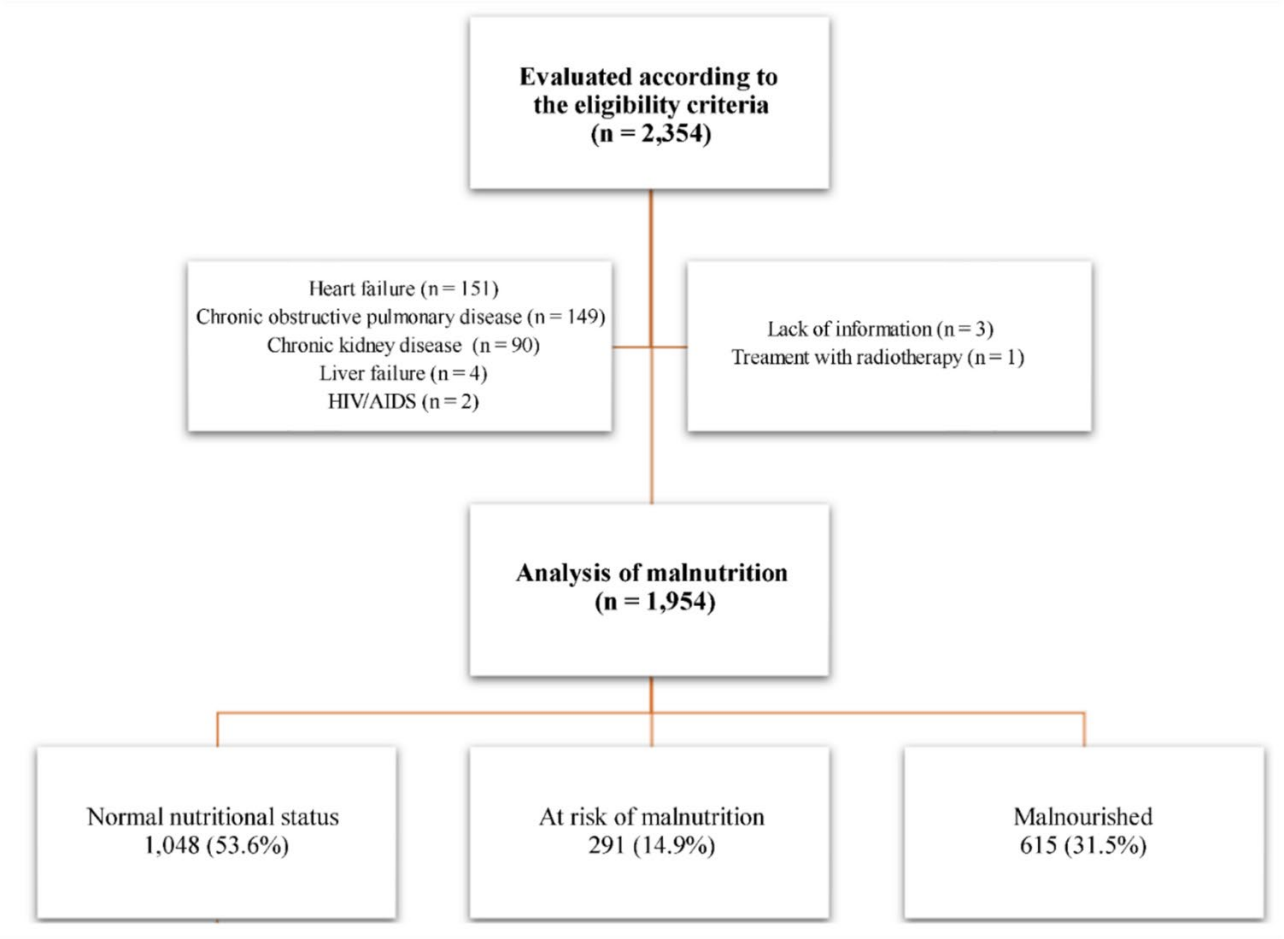
Study flowchart (2015–2020)

Of the 2354 assessed for eligibility, 1954 were included. Among those excluded, 396 had other diseases, 3 had no information regarding comorbidities, and 1 underwent radiotherapy (Fig. 1). The mean age was 72.5 years, and slightly more than half were men. Most participants had a family income above one minimum wage. Regarding lifestyle factors, a substantial proportion reported current or former smoking and alcohol use, more than half were physically inactive, and the great majority had reduced functional capacity. The most common cancer sites were the reproductive system and breast, while metastasis was present in approximately one-quarter of the sample (Table 1).

**Table 1:** Sociodemographic, lifestyle, and clinical characteristics of older adults with cancer. Recife, PE, 2015–2020

Parameters	
Sociodemographic	
Age (years)—mean ± SD	72.5 ± 7.3
Age group—*n* (%)	
< 75	1226 (62.7)
≥ 75	728 (37.3)
Sex—*n* (%)	
Female	936 (47.9)
Male	1018 (52.1)
Family income—*n* (%)	
≤ one minimum wage	622 (31.8)
> one minimum wage	1332 (68.2)
Lifestyle	
Smoking—*n* (%) (*n* = 1.940)	
Smoker or ex-smoker	1022 (52.3)
Alcohol consumption—*n* (%) (*n* = 1.940)	
Current or former alcohol user	943 (48.3)
Clinical	
Primary cancer site—*n* (%)	
Upper gastrointestinal tract	293 (14.9)
Colon, rectum, anus, and anal canal	196 (10.0)
Breast	341 (17.5)
Lung	81 (4.2)
Reproductive system	826 (42.3)
Others	217 (11.1)
Metastasis—*n* (%) (*n* = 1,741)	543 (27.8)
Functionality (KPS)—*n* (%)	
≤ 50%	244 (12.5)
> 50%	1,710 (87.5)
Nutritional status **(**MNA-SF)—*n* (%)	
Malnourished	615 (31.5)
At risk of malnutrition	291 (14.9)
Normal nutritional status	1,048 (53.6)
Level of physical activity (IPAQ)—*n* (%) (*n* = 1399)	
Sedentary	1,116 (57.1)
Active	283 (14.5)

*KPS*, Karnofsky Performance Scale; *MNA-SF*, Mini Nutritional Assessment short-form; *IPAQ*, International Physical Activity Questionnaire (short version)

According to the MNA-SF, more than half of the older adults had normal nutritional status, whereas almost one-third were classified as malnourished and a smaller proportion as at risk of malnutrition. Patients who were at risk of malnutrition or were malnourished tended to be older, physically inactive, and more likely to present reduced functional capacity, multiple comorbidities, polypharmacy, metastasis, depressive symptoms, and a history of falls (Table 2).

**Table 2:** Clinical characteristics distribution and Comprehensive Geriatric Assessment according to the nutritional status of older adults with cancer. Recife, PE, 2015–2020

Variables	Total		Malnourished		At risk of malnutrition		Normal nutritional status		*p*-value *
	*n* = 1954 (%)		*n* = 615 (31.5%)		*n* = 291 (14.9%)		*n* = 1048 (53.6%)		
Age group									0.003
< 75 years old	1.226	(62.7)	366	(29.8)	167	(13.6)	693	(56.5)	
≥ 75 years old	728	(37.3)	249	(34.2)	124	(17.0)	355	(48.8)	
Metastasis									< 0.001
Yes	543	(27.8)	202	(37.2)	140	(25.8)	201	(37.0)	
No	1.198	(61.3)	342	(28.5)	123	(10.2)	733	(61.1)	
Polypharmacy									0.011
Yes	184	(9.4)	61	(33.1)	40	(21.7)	83	(45.1)	
No	1.219	(62.4)	393	(32.2)	180	(14.7)	646	(53.0)	
History of falls									< 0.001
Yes	1.584	(81.0)	469	(29.6)	227	(14.3)	888	(56.0)	
No	360	(18.4)	142	(39.4)	62	(17.2)	156	(43.3)	
Level of physical activity (IPAQ)									< 0.001
Sedentary	1.116	(57.1)	389	(34.9)	194	(17.3)	533	(47.8)	
Active	283	(14.5)	64	(22.6)	25	(8.8)	194	(68.5)	
Comorbidities (CCI)									< 0.001
≤ 2	1.494	(76.4)	449	(30.0)	191	(12.8)	854	(57.1)	
> 2	460	(23.5)	166	(36.0)	100	(21.7)	194	(42.1)	
Functionality (KPS)									< 0.001
≤ 50	244	(12.5)	84	(34.4)	116	(47.5)	44	(18.0)	
> 50	1.710	(87.5)	531	(31.0)	175	(10.2)	1.004	(58.7)	
Risk of depression (GDS)									< 0.001
Yes	488	(25.0)	193	(39.5)	112	(22.9)	183	(37.5)	
No	915	(46.8)	261	(28.5)	108	(11.8)	546	(59.7)	

*IPAQ*, International Physical Activity Questionnaire (short version); *CCI*, Charlson Comorbidity Index; *KPS*, Karnofsky Performance Scale; *GDS*, Geriatric Depression Scale

*Pearson’s chi-square test

In the univariate analysis, several variables showed significant associations with malnutrition, including female sex, gastrointestinal and lung tumors, metastasis, physical inactivity, depressive symptoms, history of falls, multiple comorbidities, and reduced functional capacity. In the multivariate analysis, female sex, specific cancer sites (upper gastrointestinal, colorectal, and lung), metastasis, physical inactivity, depressive symptoms, and history of falls remained independently associated with malnutrition (Table 3).

**Table 3:** Factors associated with malnutrition in 1,053 older adults with cancer. Recife, PE, 2015–2020

Variables	Univariate analysis		Multivariate analysis	
	PR (95%CI)	*p*-value	aPR (95%CI)	*p-*value
Sex				
Female	1.31 (1.08–1.59)		1.28 (1.03–1.59)	
Male	1.0	0.006	1.0	0.029
Primary cancer site				
Upper gastrointestinal tract	2.33 (1.65–3.30)	< 0.001	2.39 (1.66–3.45)	< 0.001
Colon, rectum, anus, anal canal	2.44 (1.72–3.47)	< 0.001	2.54 (1.77–3.64)	< 0.001
Lung	2.30 (1.38–3.83)	0.001	2.35 (1.37–4.02)	0.002
Reproductive system	1.09 (0.81–1.49)	0.546	1.29 (0.91–1.80)	0.147
Others	0.96 (0.60–1.54)	0.859	1.10 (0.67–1.80)	0.712
Breast	1.0		1.0	
Metastasis		< 0.001		0.003
Yes	1.65 (1.34–2.02)		1.37 (1.11–1.70)	
No	1.0		1.0	
History of falls		0.004		0.042
Yes	1.40 (1.11–1.77)		1.27 (1.01–1.61)	
No	1.0		1.0	
Level of physical activity (IPAQ)		< 0.001		0.007
Sedentary	1.69 (1.28–2.22)		1.46 (1.11–1.93)	
Active	1.0		1.0	
Risk of depression (GDS)		< 0.001		0.001
Yes	1.56 (1.28–1.90)		1.42 (1.16–1.73)	
No	1.0		1.0	

*PR*, prevalence ratio; *aPR*, adjusted prevalence ratio; *95%CI*, 95% confidence interval; *IPAQ*, International Physical Activity Questionnaire (short version); *GDS*, Geriatric Depression Scale

## Discussion

According to the results, about 31.5% of the cohort was malnourished, and 14.9% were at risk of malnutrition. The risk factors associated with malnutrition were female gender, upper gastrointestinal and lung tumors, metastasis, history of falls, sedentary lifestyle, and risk of depression.

In the present study, almost half of older adults with cancer were malnourished or at risk of malnutrition upon admission for oncological treatment. These findings reinforce the recommendations of guidelines on the need for nutritional status assessment during initial evaluation since malnutrition is associated with adverse events [9, 12, 15, 17]. Moreover, nutritional support is a cost-effective intervention that may reduce mortality, treat toxicity, and improve quality of life [29–32].

Two studies also found malnutrition rates (MNA-SF) among older adults undergoing cancer treatment (33.4% and 41.9%, respectively), corroborating our results [5, 33]. This finding suggests a lack of adequate nutritional care after the diagnosis. A Brazilian cross-sectional study showed that 87.2% of hospitalized older adults with cancer were malnourished. However, the study used another evaluation tool (Patient-Generated Subjective Global Assessment), and almost half of the sample presented metastasis, which could justify this result [4].

Cancer is a disease with 60.0% of malignancy and 70.0% of deaths among older adults, which may make their healthcare challenging, especially in low-income countries (e.g., Brazil) [5, 34]. Previous studies did not evaluate the prevalence and factors associated with malnutrition in Brazilian older adults with cancer upon admission for outpatient treatment. The present study evidenced a high prevalence of malnutrition before outpatient treatment, and Costa et al. [6], using the same sample, observed a worse prognosis, in which malnutrition was associated with infection, hospitalization, and death within six months. Thus, both studies suggest a concerning association between malnutrition and prognosis.

A cohort of 608 older adults with cancer proposed a practical instrument for predicting death within 6 months based on the CGA tools. The nutritional risk and malnutrition increased the death risk. Therefore, the nutritional status assessed by MNA was suggested to be incorporated into GCA in the clinical routine of older adults with cancer [35].

### Tumor-related risks: type and disease severity

Factors that lead to nutritional risk in older adults with cancer should be investigated since they may help prevent this disorder [7]. Given the several types of tumors, healthcare professionals who are not nutritionists and have difficulty recognizing the characteristics of malnutrition must promptly identify those that most impact nutritional status. In the present study, patients with gastrointestinal tract and lung tumors had the highest risk of malnutrition, and higher rates and severity of this nutritional condition have also been observed in previous studies with people presenting these solid tumors [4, 5, 36]. In addition, a French multicentric study conducted with 1545 hospitalized adult patients with cancer demonstrated that upper gastrointestinal tumors increased the nutritional risk by almost twofold (multivariate analysis) [36]. In another Brazilian study with 277 hospitalized patients, lower gastrointestinal and lung tumors were associated with malnutrition only in the univariate analysis [4]. The difference in the results may be justified by the diagnostic criteria of malnutrition, which were different for the three studies [4, 36]. The site of the tumors increases susceptibility to treatment side effects (e.g., anorexia, diarrhea, nausea, vomiting, and dyspepsia), which impair the ingestion, digestion, assimilation, and utilization of nutrients [7, 13, 37].

Tumor severity can also influence the pathogenesis of malnutrition, increasing its risk by 37.0% in patients with metastasis. In addition, a French study with 1030 older adults with cancer in multiple stages found that 52.0% of the sample had cachexia and that metastasis increased the risk of this condition by 50.0% [38]. Anorexia is directly linked to advanced stages of cancer and malnutrition [7, 37]. This symptom affects 15.0 to 20.0% of patients at the time of diagnosis [7], and older adults with cancer in Brazil are more likely to present anorexia during hospitalization than younger patients [37]. The cause of anorexia is complex and related to acute and chronic inflammations developed from biochemical factors produced by the tumor and patient, which affect appetite regulation by the central nervous system. Additionally, these biochemical factors increase energy expenditure and muscle catabolism, which result in involuntary weight loss, one of the main signs of malnutrition [7, 13, 37].

### Functional status and physical vulnerability

In older adults, muscle mass reduction occurs due to changes in the constitution and architecture of skeletal muscle, anorexia related to aging, metabolic effects, inflammatory diseases, and inactivity [1]. Although most of the sample of this study presented adequate functionality, more than half were sedentary, and this condition increased the risk of malnutrition by 50.0% due to difficulties in gaining and maintaining muscle and bone mass. Moreover, the sedentary lifestyle rate found in this study exceeded the Brazilian mean for the older population [39, 40].

Most patients had a history of falls in the last year. More than 40.0% who had fallen presented worse nutritional status, and the risk of becoming malnourished increased by 27.0%, which can be explained by mobility and physical performance impairment, hampering daily activities (e.g., cooking and eating) [7]. In the present study, functionality was not a risk factor for malnutrition, contradicting previous studies [3, 4, 33, 36]. However, functional capacity may also have been affected since it was associated with a poorer nutritional status.

### Depression and nutrition-impact symptoms

Depression and other neuropsychological disorders may increase nutritional risk in patients with cancer [7, 41]. The symptoms of depression could impact nutrition, leading to appetite loss, a lack of interest in leisure activities, and reduced food intake [7, 41]. Similar to this study, depression was highly prevalent in older adults with cancer [3, 33, 38, 42] and was a risk factor for malnutrition [33] and cachexia [38]. Therefore, educating healthcare professionals on mental disorders must be a focus to improve the recognition of depression and anxiety symptoms in this population [7, 33, 41].

### Sex differences in malnutrition risk

According to our results, being female increased the likelihood of malnutrition by nearly 30%. This association may be explained by the fact that women often present a higher prevalence of nutrition-impact symptoms related to food intake, such as loss of appetite and reduced dietary consumption, compared with men across different clinical conditions [43]. Additionally, this finding may have been influenced by the high proportion of breast cancer in the sample, similar to a cross-sectional study in which breast cancer patients were 2.60 times more likely to be malnourished than those with other tumor types, due to the higher prevalence of weight loss and low BMI at hospital admission [44].

Other nutrition-impact symptoms relevant to the development of malnutrition, such as anorexia, nausea, and xerostomia, may also have contributed to this result, particularly among women with breast cancer, consistent with findings from a Brazilian multicenter study [5].

This study has some limitations that should be considered. As a cross-sectional study, it presents inherent limitations of this design, particularly the inability to establish cause-effect relationships. Additionally, the use of secondary data may result in missing information for some variables; however, these limitations were minimized by the prospective data collection of the original study, which followed a standardized and rigorous protocol, as well as by the large sample size and the careful procedures used for data collection, verification, and standardization. Moreover, there was no monitoring of changes in nutritional status over the course of the original study’s follow-up period, which prevented longitudinal analyses and the assessment of clinical factors related to changes in nutritional status.

This study offers significant contributions that reinforce its relevance to the care of older adults with cancer. The investigation includes a large and representative sample evaluated at a reference center in Northeast Brazil, allowing for a comprehensive clinical and nutritional profile of these patients at the beginning of oncological treatment, based on tools of the CGA. Although this is a secondary analysis, the original data were collected prospectively, and the main anthropometric measurements were obtained at the time of evaluation by trained professionals, minimizing biases commonly associated with this type of approach.

The use of the MNA-SF also stands out for its practicality and low cost, in addition to being a validated and sensitive tool, particularly suitable for outpatient settings with high patient demand and limited resources. Another strength of the study is its methodological rigor, which included precise variable definitions and multivariate analysis capable of identifying independent factors associated with malnutrition. Taken together, these aspects provide high clinical applicability and contribute to advancing knowledge on nutritional assessment and comprehensive care for older adults with cancer in outpatient settings.

Malnutrition and nutritional risk were highly prevalent among older adults at the beginning of cancer treatment in Northeast Brazil. The factors associated with malnutrition included female sex, gastrointestinal and lung tumors, metastatic disease, history of falls, physical inactivity, and risk of depression. These findings indicate that such conditions should serve as warning signs for the potential deterioration of nutritional status in this population. Considering that compromised nutritional status is associated with poorer clinical outcomes in older adults with cancer, it is imperative that oncology care centers prioritize nutritional assessment upon patient admission. When nutritional risk or malnutrition is identified, patients should be promptly referred for specialized nutritional follow-up to ensure appropriate management.

We recommend that future studies adopt longitudinal designs, include older adults with cancer at different phases of oncological treatment, and conduct nutritional intervention research to more robustly evaluate the effects of nutritional care on clinical outcomes in this population.

## Data Availability

No datasets were generated or analysed during the current study.
